# Supplementary material for: Capturing a Crucial ‘Disorder-to-Order Transition’ at the Heart of the Coronavirus Molecular Pathology—Triggered by Highly Persistent, Interchangeable Salt-Bridges
Source: Vaccines (Basel). 2022 Feb 16;10(2):301. doi: 10.3390/vaccines10020301 (PMC8875383; doi:10.3390/vaccines10020301)
Supplement: Supplementary file 1 [file vaccines-10-00301-s001.zip › vaccines-1555047-supplementary.pdf]

Supplementary Materials for the paper

Capturing a crucial 'disorder-to-order transition' at the heart of the coronavirus molecular pathology – triggered by highly persistent, interchangeable salt-bridges

by

Sourav Roy, Prithwi Ghosh, Abhirup Bandyopadhyay, Sankar Basu[*]

Content: *10 Supplementary Figures, 8 Supplementary Tables, and 1 Supplementary Video (Link & caption).*

Table S1. Dataset of representative Coronavirus (CoV/CoV-2) Spike experimental (cryo-EM) structures with a resolution not worse than 3 Å, extracted from the PDB. The title description extracted from the PDB headers are given for each entry, along with their cryo-EM resolutions (Å) and coronavirus lineages (CoV/CoV-2).

PDB ID	Resolution (Å)	Description (TITLE)	CoV/CoV-2
6m15	2.38	Cryo-Em Structures Of Hku2 Spike Glycoproteins	CoV
6m16	2.83	Cryo-Em Structures Of Sads-Cov Spike Glycoproteins	CoV
6nzk	2.80	Structural Basis For Human Corona Virus Attachment To Sialic Acid Receptors	CoV
6ohw	2.90	Structural Basis For Human Corona Virus Attachment To Sialic Acid Receptors. Apo-Hcov-Oc43 S	CoV
6q04	2.50	Mers-Cov S Structure In Complex With 5-N-Acetyl Neuraminic Acid	CoV
6q05	2.80	Mers-Cov S Structure In Complex With Sialyl-Lewisx	CoV
6q06	2.70	Mers-Cov S Structure In Complex With 2,3-Rialyl-N-Acetyl-Lactosamine	CoV
6q07	2.90	Mers-Cov S Structure In Complex With 2,6-Rialyl-N-Acetyl-Lactosamine	CoV
7bbh	2.90	Structure Of Coronavirus Spike From Smuggled Guangdong Pangolin	CoV
7cn4	2.93	Cryo-Em Structure Of Bat Ratg13 Spike Glycoprotein	CoV
7cn8	2.50	Cryo-Em Structure Of Pcov_Gx Spike Glycoprotein	CoV
7m5e	2.50	Mers-Cov S Bound To The Broadly Neutralizing B6 Fab Fragment (C3 Refinement)	CoV
6vxx	2.80	Structure Of The Sars-Cov-2 Spike Glycoprotein (CLOSED State)	CoV-2
6x29	2.70	Sars-Cov-2 Rs2d Down State Spike Protein Trimer	CoV-2
6x79	2.90	Prefusion Sars-Cov-2 S Ectodomain Trimer Covalently Stabilized In The Closed Conformation	CoV-2
6xlu	2.40	Structure Of Sars-Cov-2 Spike At Ph 4.0	CoV-2
6xm0	2.70	Consensus Structure Of Sars-Cov-2 Spike At Ph 5.5	CoV-2
6xm3	2.90	Structure Of Sars-Cov-2 Spike At Ph 5.5, Single Rbd Up, Conformation 1	CoV-2

6xm4	2.90	Structure Of Sars-Cov-2 Spike At Ph 5.5, Single Rbd Up, Conformation 2	CoV-2
6xr8	2.90	Distinct Conformational States Of Sars-Cov-2 Spike Protein	CoV-2
6xra	3.00	Distinct Conformational States Of Sars-Cov-2 Spike Protein	CoV-2
6zb5	2.85	Sars Cov-2 Spike Protein, Closed Conformation, C3 Symmetry	CoV-2
6zge	2.60	Uncleavable Spike Protein Of Sars-Cov-2 In Closed Conformation	CoV-2
6zgi	2.90	Furin Cleaved Spike Protein Of Sars-Cov-2 In Closed Conformation	CoV-2
6zow	3.00	Sars-Cov-2 Spike In Prefusion State	CoV-2
6zox	3.00	Structure Of Disulphide-Rtabilized Sars-Cov-2 Spike Protein Trimer (X2 Disulphide-Bond Mutant, G413c, V987c, Single Arg S1/S2 Cleavage Site)	CoV-2
6zp0	3.00	Structure Of Sars-Cov-2 Spike Protein Trimer (SINGLE Arg S1/S2 Cleavage Site) In Closed State	CoV-2
7a4n	2.75	Cryo-Em Structure Of A Prefusion Stabilized Sars-Cov-2 Spike (D614N, R682s, R685g, A892p, A942p And V987p)(S-Closed Trimer)	CoV-2
7ad1	2.92	Cryo-Em Structure Of A Prefusion Stabilized Sars-Cov-2 Spike (D614N, R682s, R685g, A892p, A942p And V987p)(One Up Trimer)	CoV-2
7ddd	3.00	Sars-CoV-2 S Protein At Close State	CoV-2
7df3	2.70	Sars-Cov-2 S Trimer, S-Closed	CoV-2
7dwy	2.70	S Protein Of Sars-Cov-2 In The Locked Conformation	CoV-2
7e7b	2.60	Cryo-Em Structure Of The Sars-Cov-2 Furin Site Mutant S-Trimer From A Subunit Vaccine Candidate	CoV-2
7jwy	2.50	Structure Of Sars-Cov-2 Spike At Ph 4.5	CoV-2
7kdk	2.80	Sars-Cov-2 D614g 3 Rbd Down Spike Protein Trimer Without The P986-P987 Stabilizing Mutations (S-GSAS-D614G)	CoV-2
7kdl	2.96	Sars-Cov-2 D614g 1-Rbd Up Spike Protein Trimer Without The P986-P987 Stabilizing Mutations (S-GSAS-D614G)	CoV-2
7lwk	2.92	Mink Cluster 5-Associated Sars-Cov-2 Spike Protein (S-GSAS-D614G-DELFV) In The 3-Rbd Down Conformation	CoV-2
7lwl	2.84	Mink Cluster 5-Associated Sars-Cov-2 Spike Protein (S-GSAS-D614G-DELFV) In The 3-Rbd Down Conformation	CoV-2
7lwm	2.83	Mink Cluster 5-Associated Sars-Cov-2 Spike Protein (S-GSAS-D614G-DELFV) In The 1-Rbd Up	CoV-2

		Conformation	
7lwn	2.94	Mink Cluster 5-Associated Sars-Cov-2 Spike Protein (S-GSAS-D614G-DELFV) In The 1-Rbd Up Conformation	CoV-2
7lwo	2.85	Mink Cluster 5-Associated Sars-Cov-2 Spike Protein (S-GSAS-D614G-DELFV) In The 1-Rbd Up Conformation	CoV-2
7lww	3.00	Triple Mutant (K417N-E484K-N501Y) Sars-Cov-2 Spike Protein In The 1-Rbd-Up Conformation (S-GSAS-D614G-K417N-E484K-N501Y)	CoV-2
7mjg	2.81	Cryo-Em Structure Of The Sars-Cov-2 N501y Mutant Spike Protein Ectodomain	CoV-2
7mtc	2.60	Structure Of Freshly Purified Sars-Cov-2 S2p Spike At Ph 7.4	CoV-2

Figures S1. Pairwise alignments of the FLCS$_{\text{Spike}}$ sequences extracted from 7AKJ (SARS-CoV Spike) and 6XR8 (SARS-CoV-2 Spike). Sources of these sequence data are abbreviated (PM, EM) as mentioned in the legend box. The activation loop motifs ('PRRAR' in CoV-2 and its aligned homologous sequence in CoV) are highlighted in bold with distinct font and background colors. Sequences derived from proteomic data (PM) that are found missing for their experimental structural patches (EM) are demarcated with distinct background colors in CoV, CoV-2 for the regions flanking to the activation loop motifs. As is standard, a '-' refers to a gap (i.e., missing residue) in the corresponding alignments.

```
>7akj_EM   ASYHTV-------QKSIVAYTMSLGADSSIAYSNNTIAIPTNFSISITTEVMPVSM       720
>7akj_PM   ASYHTVSLLRSTSQKSIVAYTMSLGADSSIAYSNNTIAIPTNFSISITTEVMPVSM       720

>6xr8_EM   ASYQT------------SQSIIAYTMSLGAENSVAYSNNSIAIPTNFTISVTTEILPVSM   720
>6xr8_PM   ASYQTQTNSPRRARSVASQSIIAYTMSLGAENSVAYSNNSIAIPTNFTISVTTEILPVSM   720

>7akj_PM   ASYHTVS----LLRSTSQKSIVAYTMSLGADSSIAYSNNTIAIPTNFSISITTEVMPVSM   720
>6xr8_PM   ASYQTQTNSPRRARSVASQSIIAYTMSLGAENSVAYSNNSIAIPTNFTISVTTEILPVSM   720

PM: Proteomic (Sequence) Data
EM: Electron Microscopic (Structural) Data

7akj: SARS-CoV  (2003) Spike
6xr8: SARS-CoV2 (2019) Spike
```

Figures S2. Multiple Sequence alignment (MSA) of the coronavirus FLCS$_{Spike}$ sequences with the unique pentapeptide activation loops highlighted (colored differentially). MSAs were performed by MUSCLE [1].

```
6m15    NPLGDGFCADLLSNVVV---------RRMTFEKHDTTY-------VAPVTNERFTELPLD  569
6m16    NPLGDGFCADLLGNVAV---------RRMTFEKHDTTY-------VAPVTNERYTEMPLD  571
6nzk    LTVGSGYCVDYSKNGGSGGA----ITTGYRFTNFEPFTVNSVNDSLEPVGGLYEIQIPSE  805
6ohw    LTVGSGYCVDYSKNGGSGGA----ITTGYRFTNFEPFTVNSVNDSLEPVGGLYEIQIPSE  805
7bbh    IPIGAGICASYQTNTNS-----RSVSSQ---AII-AYTM-SLGAENSVAYANNSIAIPTN  709
7cn8    IPVGAGICASYHSMS-----SLRSVNQR---SII-AYTM-SLGAENSVAYSNNSIAIPTN  711
6zox    IPIGAGICASYQTQTNS-R----SVASQ---SII-AYTM-SLGAENSVAYSNNSIAIPTN  704
6zp0    IPIGAGICASYQTQTNS-R----SVASQ---SII-AYTM-SLGAENSVAYSNNSIAIPTN  704
6zge    IPIGAGICASYQTQTNS-PSRASSVASQ---SII-AYTM-SLGAENSVAYSNNSIAIPTN  748
6q04    LPLGQSLCALPDTPSTLTPASVGSVPGEMRLASI-AFNH-PIQV-DQLNSSYFKLSIPTN  799
6q05    LPLGQSLCALPDTPSTLTPASVGSVPGEMRLASI-AFNH-PIQV-DQLNSSYFKLSIPTN  799
6q06    LPLGQSLCALPDTPSTLTPASVGSVPGEMRLASI-AFNH-PIQV-DQLNSSYFKLSIPTN  799
6q07    LPLGQSLCALPDTPSTLTPASVGSVPGEMRLASI-AFNH-PIQV-DQLNSSYFKLSIPTN  799
7m5e    LPLGQSLCALPDTPSTLTPASVGSVPGEMRLASI-AFNH-PIQV-DQLNSSYFKLSIPTN  799
6vxx    IPIGAGICASYQTQTNS-PSGAGSVASQ---SII-AYTM-SLGAENSVAYSNNSIAIPTN  736
7mtc    IPIGAGICASYQTQTNS-PSGAGSVASQ---SII-AYTM-SLGAENSVAYSNNSIAIPTN  736
6x79    IPIGAGICASYQTQTNS-PSGAGSVASQ---SII-AYTM-SLGAENSVAYSNNSIAIPTN  736
7a4n    IPIGAGICASYQTQTNS-PSRAGSVASQ---SII-AYTM-SLGAENSVAYSNNSIAIPTN  717
7ad1    IPIGAGICASYQTQTNS-PSRAGSVASQ---SII-AYTM-SLGAENSVAYSNNSIAIPTN  717
7lwk    IPIGAGICASYQTQTNS-PGSASSVASQ---SVI-AYTM-SLGAENSVAYSNNSIAIPTN  715
7lwl    IPIGAGICASYQTQTNS-PGSASSVASQ---SVI-AYTM-SLGAENSVAYSNNSIAIPTN  715
7lwm    IPIGAGICASYQTQTNS-PGSASSVASQ---SVI-AYTM-SLGAENSVAYSNNSIAIPTN  715
7lwn    IPIGAGICASYQTQTNS-PGSASSVASQ---SVI-AYTM-SLGAENSVAYSNNSIAIPTN  715
7lwo    IPIGAGICASYQTQTNS-PGSASSVASQ---SVI-AYTM-SLGAENSVAYSNNSIAIPTN  715
7kdk    IPIGAGICASYQTQTNS-PGSASSVASQ---SII-AYTM-SLGAENSVAYSNNSIAIPTN  717
7kdl    IPIGAGICASYQTQTNS-PGSASSVASQ---SII-AYTM-SLGAENSVAYSNNSIAIPTN  717
7lww    IPIGAGICASYQTQTNS-PGSASSVASQ---SII-AYTM-SLGAENSVAYSNNSIAIPTN  717
7mjg    IPIGAGICASYQTQTNS-PGSASSVASQ---SII-AYTM-SLGAENSVAYSNNSIAIPTN  717
6x29    IPIGAGICASYQTQTNS-PGSASSVASQ---SII-AYTM-SLGAENSVAYSNNSIAIPTN  702
7df3    IPIGAGICASYQTQTNS-PGSASSVASQ---SII-AYTM-SLGAENSVAYSNNSIAIPTN  717
7ddd    IPIGAGICASYQTQTNS-PGSASSVASQ---SII-AYTM-SLGAENSVAYSNNSIAIPTN  717
6xlu    IPIGAGICASYQTQTNS-PGSASSVASQ---SII-AYTM-SLGAENSVAYSNNSIAIPTN  704
6xm0    IPIGAGICASYQTQTNS-PGSASSVASQ---SII-AYTM-SLGAENSVAYSNNSIAIPTN  704
6xm3    IPIGAGICASYQTQTNS-PGSASSVASQ---SII-AYTM-SLGAENSVAYSNNSIAIPTN  704
6xm4    IPIGAGICASYQTQTNS-PGSASSVASQ---SII-AYTM-SLGAENSVAYSNNSIAIPTN  704
6zow    IPIGAGICASYQTQTNS-PGSASSVASQ---SII-AYTM-SLGAENSVAYSNNSIAIPTN  717
7jwy    IPIGAGICASYQTQTNS-PGSASSVASQ---SII-AYTM-SLGAENSVAYSNNSIAIPTN  704
6zb5    IPIGAGICASYQTQTNS-PRRAASVASQ---SII-AYTM-SLGAENSVAYSNNSIAIPTN  717
6xr8    IPIGAGICASYQTQTNS-PRRARSVASQ---SII-AYTM-SLGAENSVAYSNNSIAIPTN  717
6xra    IPIGAGICASYQTQTNS-PRRARSVASQ---SII-AYTM-SLGAENSVAYSNNSIAIPTN  717
6zgi    IPIGAGICASYQTQTNS-PRRARSVASQ---SII-AYTM-SLGAENSVAYSNNSIAIPTN  748
7dwy    IPIGAGICASYQTQTNS-PRRARSVASQ---SII-AYTM-SLGAENSVAYSNNSIAIPTN  717
          :*  .  *.                                          :*  :
```

Figure S3. The unfiltered unsorted Furin – docked (Cluspro 2.0) ensemble of SARS-CoV-2 Spike. The figure shows Cluspro 2.0 – returned docked poses for a single run of ‘ensemble (blind) docking’ of Furin (ligand) and one of the SARS-CoV-2 Spike models (receptor) sampled from the CoV-2 $FLCS_{Spike}$ disordered ensemble.

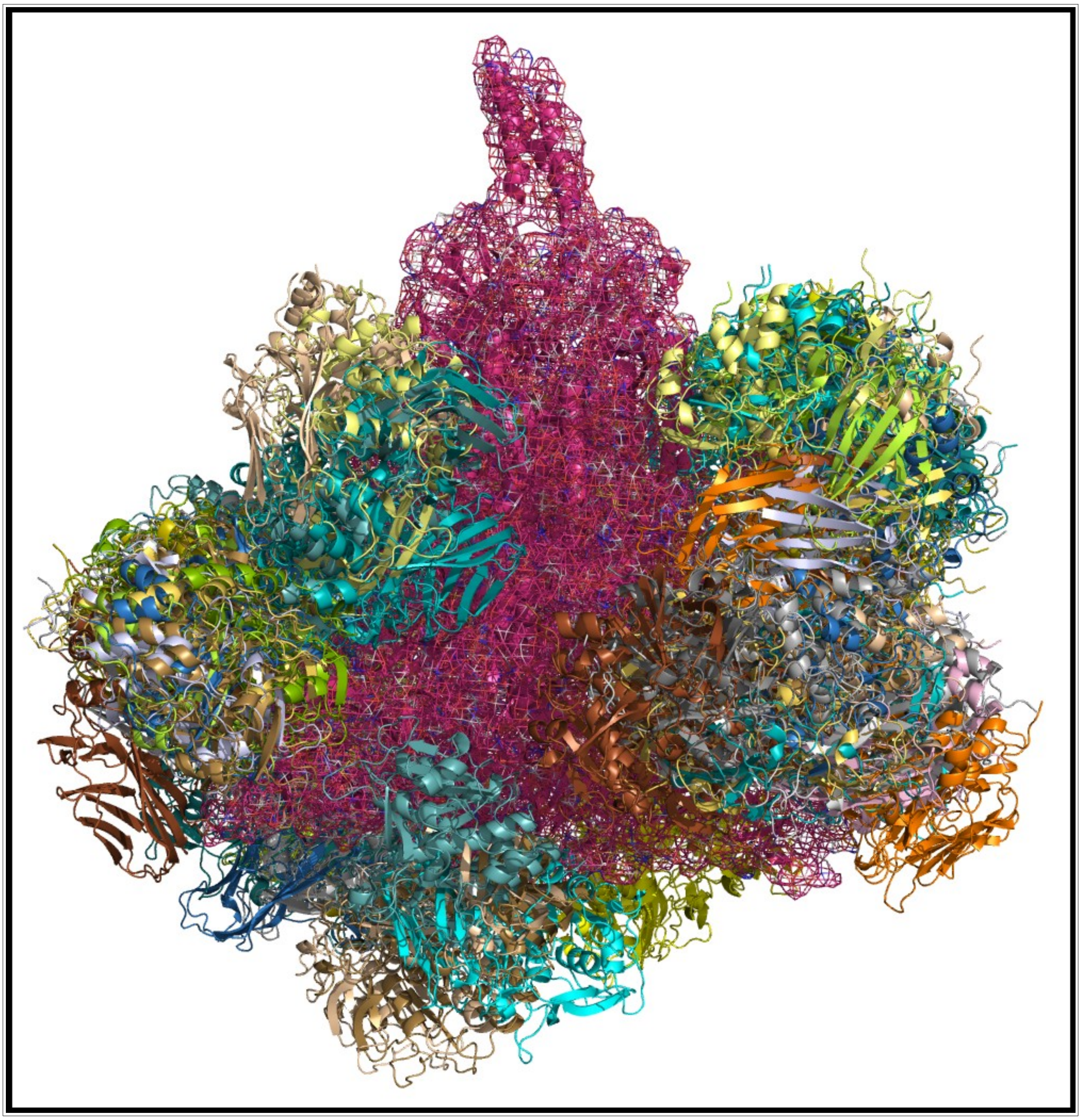

Table S2. Scores for top 100 CoV-2 Spike–Furin docked poses ranked by S_{dock}. Buried surface area (raw and normalized) for the 'PRRAR' activation loop (BSA_{PRRAR}, $nBSA_{PRRAR}$) along with the shape complementarity estimates of the FLCS patch (Sc^{FLCS}) for each docked pose are tabulated with their S_{dock} scores (for definitions, see section **2.3.2.1 - 2.3.2.3, Materials and Methods**) – based on which they were re-ranked.

Model ID (based on rank)	BSA_{PRRAR} (Å^2)	$nBSA_{PRRAR}$	Sc^{FLCS}	S_{dock}
RR1	546.128	0.247	0.770	0.972
RR2	467.328	0.241	0.696	0.906
RR3	544.543	0.210	0.716	0.876
RR4	568.184	0.216	0.691	0.864
RR5	435.363	0.228	0.661	0.858
RR6	457.662	0.238	0.631	0.852
RR7	569.994	0.191	0.712	0.847
RR8	391.570	0.189	0.711	0.843
RR9	533.784	0.221	0.642	0.834
RR10	517.731	0.146	0.755	0.832
RR11	366.049	0.156	0.730	0.821
RR12	384.993	0.150	0.733	0.816
RR13	419.974	0.196	0.667	0.816
RR14	384.423	0.195	0.668	0.816
RR15	430.356	0.120	0.761	0.814
RR16	540.292	0.198	0.657	0.811
RR17	339.607	0.162	0.710	0.810
RR18	203.347	0.076	0.784	0.805
RR19	428.685	0.191	0.661	0.805
RR20	354.425	0.138	0.727	0.799
RR21	552.749	0.119	0.741	0.795
RR22	406.678	0.106	0.752	0.794
RR23	467.913	0.160	0.688	0.788
RR24	143.555	0.066	0.771	0.787
RR25	348.837	0.114	0.737	0.787
RR26	475.195	0.189	0.637	0.782
RR27	297.038	0.106	0.739	0.782
RR28	480.299	0.190	0.635	0.782
RR29	368.961	0.119	0.726	0.780

RR30	319.929	0.102	0.739	0.778
RR31	532.620	0.203	0.603	0.776
RR32	152.871	0.059	0.755	0.768
RR33	497.013	0.147	0.673	0.760
RR34	452.581	0.151	0.667	0.760
RR35	415.054	0.141	0.678	0.759
RR36	654.652	0.175	0.626	0.754
RR37	327.674	0.068	0.734	0.752
RR38	456.972	0.145	0.664	0.750
RR39	335.308	0.142	0.668	0.750
RR40	567.676	0.180	0.613	0.750
RR41	524.557	0.199	0.575	0.749
RR42	486.607	0.192	0.589	0.748
RR43	221.766	0.067	0.728	0.746
RR44	382.312	0.122	0.685	0.745
RR45	498.099	0.224	0.515	0.745
RR46	315.677	0.124	0.682	0.744
RR47	389.157	0.096	0.706	0.743
RR48	422.396	0.107	0.691	0.738
RR49	479.041	0.237	0.467	0.736
RR50	561.150	0.158	0.629	0.735
RR51	384.495	0.137	0.651	0.730
RR52	211.027	0.089	0.697	0.729
RR53	213.287	0.065	0.710	0.727
RR54	278.004	0.123	0.663	0.726
RR55	556.757	0.169	0.599	0.724
RR56	345.334	0.169	0.599	0.724
RR57	324.116	0.132	0.646	0.719
RR58	617.661	0.135	0.638	0.716
RR59	241.915	0.071	0.692	0.713
RR60	268.053	0.089	0.676	0.709
RR61	312.872	0.097	0.668	0.707
RR62	277.198	0.122	0.641	0.705
RR63	378.888	0.136	0.624	0.704
RR64	327.364	0.069	0.682	0.702
RR65	398.884	0.151	0.598	0.700
RR66	533.770	0.192	0.517	0.693

RR67	338.996	0.111	0.639	0.693
RR68	312.146	0.139	0.602	0.688
RR69	411.373	0.131	0.608	0.685
RR70	358.715	0.135	0.598	0.681
RR71	253.788	0.090	0.643	0.678
RR72	404.504	0.102	0.628	0.674
RR73	209.889	0.055	0.660	0.673
RR74	282.161	0.094	0.631	0.670
RR75	222.060	0.052	0.657	0.669
RR76	279.145	0.066	0.648	0.667
RR77	340.124	0.078	0.640	0.667
RR78	237.750	0.056	0.652	0.666
RR79	313.640	0.094	0.627	0.666
RR80	273.857	0.061	0.648	0.664
RR81	273.857	0.061	0.648	0.664
RR82	217.383	0.055	0.649	0.662
RR83	455.640	0.152	0.551	0.661
RR84	237.896	0.067	0.638	0.658
RR85	239.638	0.057	0.644	0.658
RR86	207.618	0.053	0.645	0.657
RR87	410.788	0.096	0.613	0.655
RR88	310.860	0.086	0.620	0.654
RR89	326.202	0.085	0.619	0.652
RR90	294.880	0.081	0.618	0.648
RR91	241.056	0.051	0.627	0.639
RR92	313.072	0.082	0.606	0.637
RR93	309.123	0.099	0.590	0.636
RR94	352.503	0.105	0.584	0.636
RR95	394.385	0.111	0.571	0.631
RR96	266.771	0.068	0.603	0.625
RR97	325.878	0.151	0.508	0.624
RR98	378.979	0.097	0.573	0.619
RR99	309.579	0.082	0.584	0.616
RR100	309.579	0.082	0.584	0.616

Table S3. Furin anionic residues (Asp, Glu) having the potential to form interfacial salt-bridges with FLCS$_{\mathbf{Spike}}$. Exposed (or partially exposed) anionic residues proximal to the Furin catalytic triad are highlighted with font color 'purple'.

Residues	D_{triad}	*bur*	Burial status
153-ASP	3.264	0.00	buried
162-ASP	17.999	0.00	buried
157-GLU	14.000	0.01	buried
201-GLU	9.627	0.01	buried
331-GLU	15.068	0.01	buried
154-ASP	5.707	0.04	buried
174-ASP	15.288	0.04	buried
168-ASP	19.224	0.06	partially exposed
236-GLU	14.153	0.08	partially exposed
301-ASP	16.911	0.08	partially exposed
306-ASP	14.002	0.08	partially exposed
355-ASP	11.346	0.12	partially exposed
228-ASP	11.503	0.15	partially exposed
233-ASP	19.726	0.24	partially exposed
259-ASP	17.834	0.26	partially exposed
299-GLU	18.572	0.28	partially exposed
179-ASP	19.908	0.31	exposed
258-ASP	11.858	0.31	exposed
181-ASP	18.167	0.37	exposed
362-GLU	18.798	0.42	exposed
264-ASP	17.833	0.46	exposed
191-ASP	11.772	0.56	exposed
177-ASP	19.117	0.57	exposed
257-GLU	14.321	0.64	exposed
230-GLU	18.719	0.72	exposed

Table S4. Occurrence and average contact intensities of all unique salt-bridges at the SARS-CoV-2 Spike–Furin interface computed on the static ensemble of the top 100 (re-)ranked docked poses (as enlisted in Table S2). '-S' & '-F' in the salt-bridge descriptor strings refer to the receptor and the ligand chains respectively. Rows corresponding to the arginine – salt-bridges falling within the pentapeptide $_{681}$PRRAR$_{685}$ motif (activation loop of FLCS$_{Spike}$) is highlighted in three different font colors for R682 (red), R683 (purple), R685 (green).

Salt-bridge	***TotC***	***Frames$_p$***	***ACI***	***Occ***
214-ARG-S ↔ 112-GLU-F	*2*	*1*	*2.00*	*0.01*
654-GLU-S ↔ 357-ARG-F	*4*	*1*	*4.00*	*0.01*
654-GLU-S ↔ 193-ARG-F	*6*	*2*	*3.00*	*0.02*
682-ARG-S ↔ 233-ASP-F	*7*	*3*	*2.33*	*0.03*
683-ARG-S ↔ 230-GLU-F	*5*	*3*	*1.67*	*0.03*
685-ARG-S ↔ 306-ASP-F	*11*	*6*	*1.83*	*0.06*
682-ARG-S ↔ 236-GLU-F	*26*	*11*	*2.36*	*0.11*
685-ARG-S ↔ 236-GLU-F	*46*	*17*	*2.71*	*0.17*
682-ARG-S ↔ 230-GLU-F	*52*	*18*	*2.89*	*0.18*
683-ARG-S ↔ 236-GLU-F	*57*	*25*	*2.28*	*0.25*
683-ARG-S ↔ 264-ASP-F	*64*	*28*	*2.29*	*0.28*
685-ARG-S ↔ 264-ASP-F	*86*	*31*	*2.77*	*0.31*

TotC: *Total Counts (Ion-pairs)*
Frames$_p$: *Number of frames the salt-bridge (Residue-Pair) is found in*
ACI: *Average Contact Intensity = TotC/Frames$_p$*
Occ: *Occurrence = Frames$_p$/Frames$_t$*
Frames$_t$=Total number of models in the static ensemble: 100

Figure S4. The reference Spike – Furin ensemble (static) comprising of plausible Furin poses docked onto Spike with the selected top ranked ($RR1_{CoV-2}$) docked pose highlighted. The ensemble consisted of 100 top ranked static docked poses while only the top 50 re-ranked poses have been displayed for visual clarity. In the displayed superposed ensemble-docked poses, $RR1_{CoV-2}$ (static) is highlighted by blue dots while its $FLCS_{Spike}$ is highlighted in red. The inset displays the full ionic bond network (i.e., all Spike – Furin interfacial salt-bridges to have occurred) of $RR1_{CoV-2}$ (Furin: blue cartoon, $FLCS_{Spike}$: ivory) with the salt-bridge forming charged residues labeled with their residues identities, highlighted in the background of other static poses (cyan cartoons).

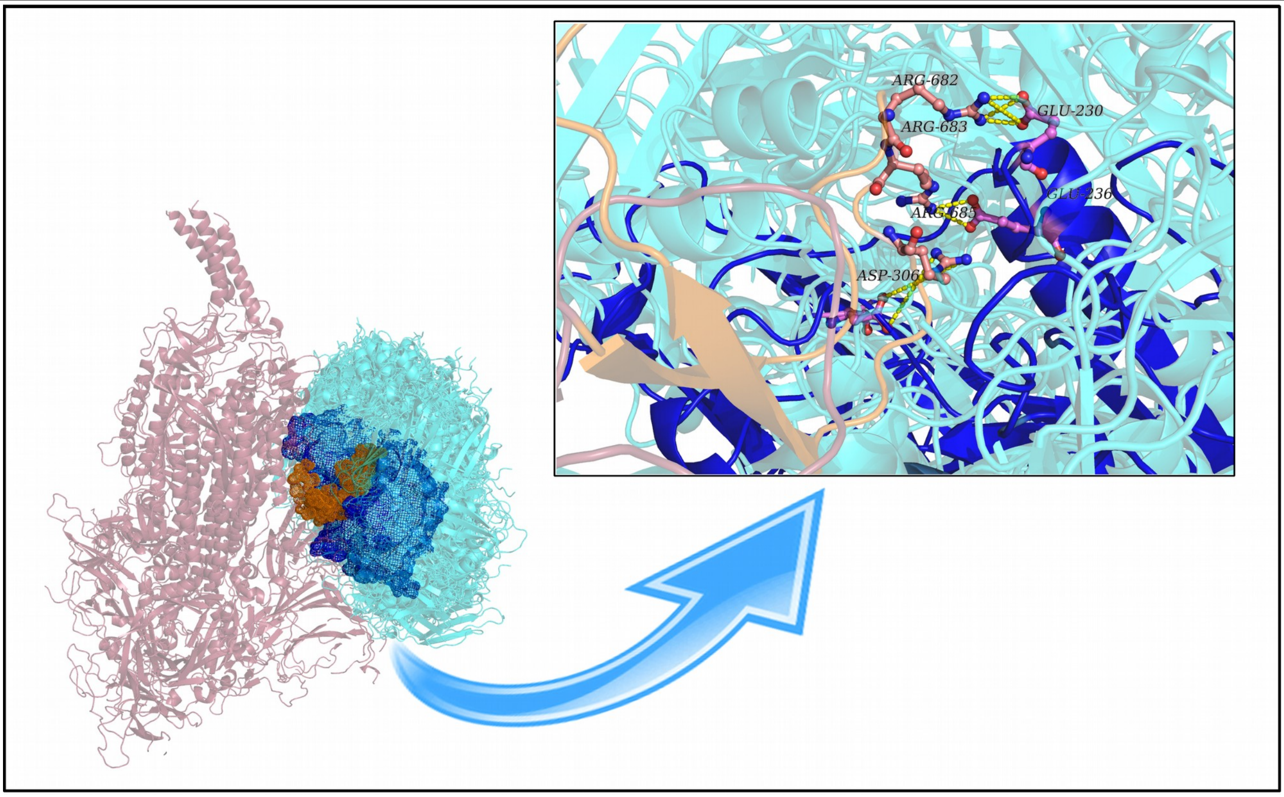

Video S1. The Spike–Furin binding in SARS-CoV-2: Essential Dynamics of Interfacial Salt-bridges portrayed (see ‘*unlisted*’ YouTube link: https://www.youtube.com/watch?v=wsHKpr9gZ9E). Video built in PyMol based on a 10 ns epoch (i.e., 1000 frames, 150-160 ns) extracted from the 300 ns long trajectory of $RR1_{CoV-2}$ portraying the high persistence salt-bridges at the Spike–Furin interface in real time. The catalytic triad is shown in dots.

Table S5. Persistence and average contact intensities of all unique salt-bridges at the SARS-CoV-2 Spike–Furin interface for ZR1$_{CoV-2}$ (zdock + IraPPA re-ranking) along its 100 ns MD simulation trajectory. '-S' & '-F' in the salt-bridge descriptor strings refer to the receptor and the ligand chains respectively. Rows corresponding to the arginine – salt-bridges falling within the pentapeptide $_{681}$PRRAR$_{685}$ motif (activation loop of FLCS$_{Spike}$) is highlighted in three different font colors for R682 (red), R683 (purple), R685 (green).

Salt-bridge	*TotC*	*Frames$_p$*	*ACI*	*Pers*
683-ARG-S↔258-ASP-F	*1*	*1*	*1.00*	*0.00010*
811-LYS-S↔460-ASP-F	*2*	*2*	*1.00*	*0.00020*
685-ARG-S↔230-GLU-F	*5*	*5*	*1.00*	*0.00050*
627-ASP-S↔197-ARG-F	*8*	*8*	*1.00*	*0.00080*
825-LYS-S↔500-ASP-F	*15*	*13*	*1.15*	*0.00130*
214-ARG-S↔112-GLU-F	*22*	*18*	*1.22*	*0.00180*
683-ARG-S↔236-GLU-F	*47*	*46*	*1.02*	*0.00460*
936-ASP-S↔497-ARG-F	*130*	*83*	*1.57*	*0.00830*
214-ARG-S↔136-GLU-F	*579*	*337*	*1.72*	*0.03370*
683-ARG-S↔230-GLU-F	*524*	*405*	*1.29*	*0.04050*
685-ARG-S↔264-ASP-F	*886*	*563*	*1.57*	*0.05631*
654-GLU-S↔193-ARG-F	*962*	*567*	*1.70*	*0.05671*
627-ASP-S↔193-ARG-F	*809*	*587*	*1.38*	*0.05871*
627-ASP-S↔357-ARG-F	*3975*	*1362*	*2.92*	*0.13621*
214-ARG-S↔131-ASP-F	*4315*	*2041*	*2.11*	*0.20412*
683-ARG-S↔264-ASP-F	*4649*	*2740*	*1.70*	*0.27403*
627-ASP-S↔359-LYS-F	*4029*	*3004*	*1.34*	*0.30043*
683-ARG-S↔259-ASP-F	*9022*	*4891*	*1.84*	*0.48915*
685-ARG-S↔236-GLU-F	*33470*	*9689*	*3.45*	*0.96900*
682-ARG-S↔230-GLU-F	*38134*	*9911*	*3.85*	*0.99120*

TotC: *Total Counts (Ion-pairs)*
Frames$_p$: *Number of frames the salt-bridge (Residue-Pair) is found in*
ACI: *Average Contact Intensity = TotC/ Frames$_p$*
Pers: *Persistence = Frames$_p$/ Frames$_t$*
Frames$_t$ = Total number of frames=10000 (sampled at 10 ps interval)

Figure S5. Densely connected composite ionic bond motifs in the persistent salt-bridges formed at the Spike–Furin interface (ZR1$_{CoV-2}$). The figure plots a representative snapshot of the interface (with a close-up view for the ionic bond motifs in its inset) randomly picked from the trajectory of ZR1$_{CoV-2}$. The same from RR1$_{CoV-2}$ is portrayed in **Figure 6**.

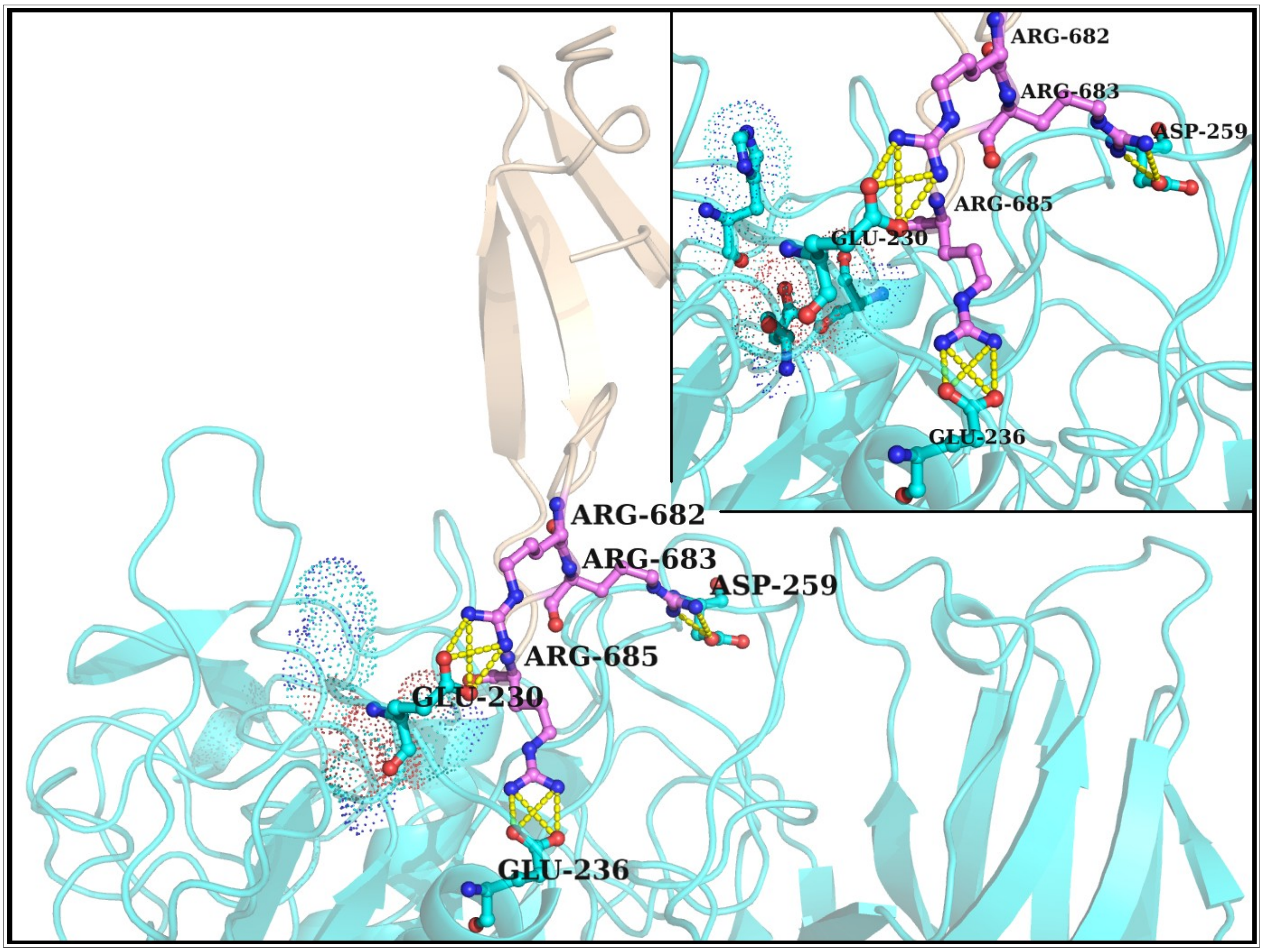

Figure S6. Frequency distributions of salt-bridge persistence and ACI in RR1$_{CoV-2}$, ZR1$_{CoV-2}$. Normalized frequencies of salt-bridges falling into each persistence bin (see section **3.6.2**) are plotted for RR1$_{CoV-2}$, ZR1$_{CoV-2}$ with different colors (as specified in the legend box) in panel A while the same for ACI are plotted in the inset of panel A. As weighted persistence (*wpers*) is the direct product of the two terms (see section **2.5.2.2, Materials and Methods**), the frequency distribution (normalized) for *wpers* is plotted in a separate panel (panel B).

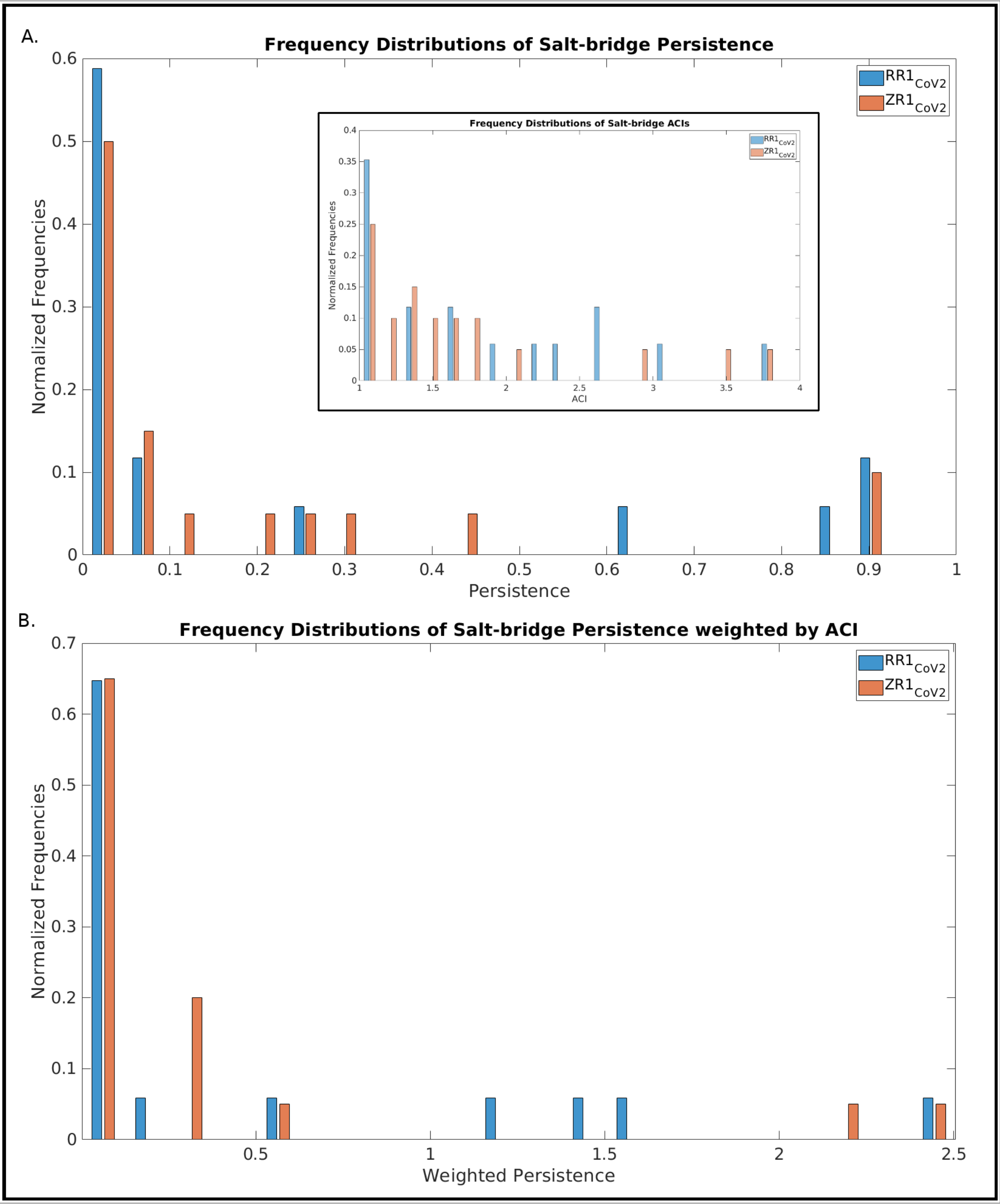

Table S6. Persistence and average contact intensities of all unique salt-bridges at the SARS-CoV Spike–Furin interface for ZR1$_{CoV}$ *(zdock+ IRAppA re-ranking)* along its 100 ns MD simulation trajectory. '-S' & '-F' in the salt-bridge descriptor strings refer to the receptor and the ligand chains respectively. Rows corresponding to high persistence interfacial salt-bridges are highlighted in different font colors unique to their cationic partners (indigo: R667, dark green: K672) coming from the SARS-CoV FLCS$_{Spike}$.

Salt-bridge	TotC	*Frames$_p$*	ACI	Pers
208-ASP-S ↔ 349-LYS-F	13	13	1.00	0.00130
294-GLU-S ↔ 298-ARG-F	1	1	1.00	0.00010
640-GLU-S ↔ 193-ARG-F	25	25	1.00	0.00250
207-ARG-S ↔ 131-ASP-F	9	9	1.00	0.00090
208-ASP-S ↔ 130-ARG-F	765	231	3.31	0.02310
667-ARG-S ↔ 258-ASP-F	2865	2787	1.03	0.27870
672-LYS-S ↔ 258-ASP-F	9167	5839	1.57	0.58390
667-ARG-S ↔ 230-GLU-F	11971	6147	1.95	0.61470
667-ARG-S ↔ 257-GLU-F	36176	9505	3.81	0.95050

TotC: *Total Counts (Ion-pairs)*
Frames$_p$: *Number of frames the salt-bridge (Residue-Pair) is found in*
ACI: *Average Contact Intensity = TotC/ Frames$_p$*
Pers: *Persistence = Frames$_p$/ Frames$_t$*
Frames$_p$ = Total number of frames=10000 (sampled at 10 ps interval)

Figure S7. Interfacial ionic bond network in ZR1$_{CoV}$ (the baseline). The figure shows the proximal looping of the FLCS$_{Spike}$ enabling R667 and K672 to be involved in multiple persistent as well as interchangeable Spike – Furin interfacial salt-bridges in SARS-CoV.

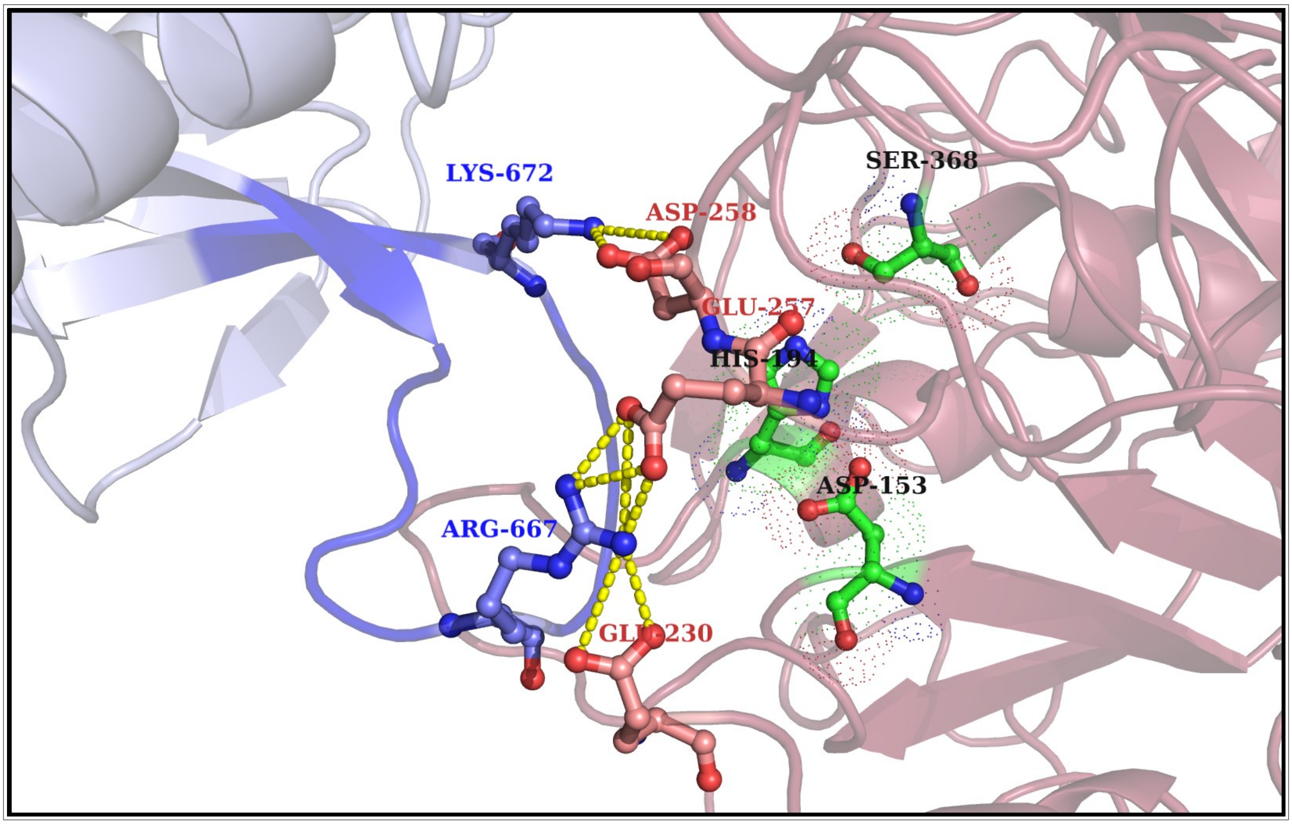

Figure S8. Interaction energy profiles (FoldX) for the top ranked Spike–Furin complexes in the cross-validation (panel A. ZR1$_{CoV-2}$) and baseline (panel B. ZR1$_{CoV}$) subjects along their respective 100 ns MD simulation trajectories. The different transition enthalpic (ΔH_{vdw}, ΔH_{elec}) and entropic ($T\Delta S_{mc}$, $T\Delta S_{sc}$) terms along with the net $\Delta G_{binding}$ are plotted in different colors as has been mentioned in the corresponding legend-boxes. All thermodynamic parameters are essentially energy terms and are plotted in the units of kcal mol^{-1}.

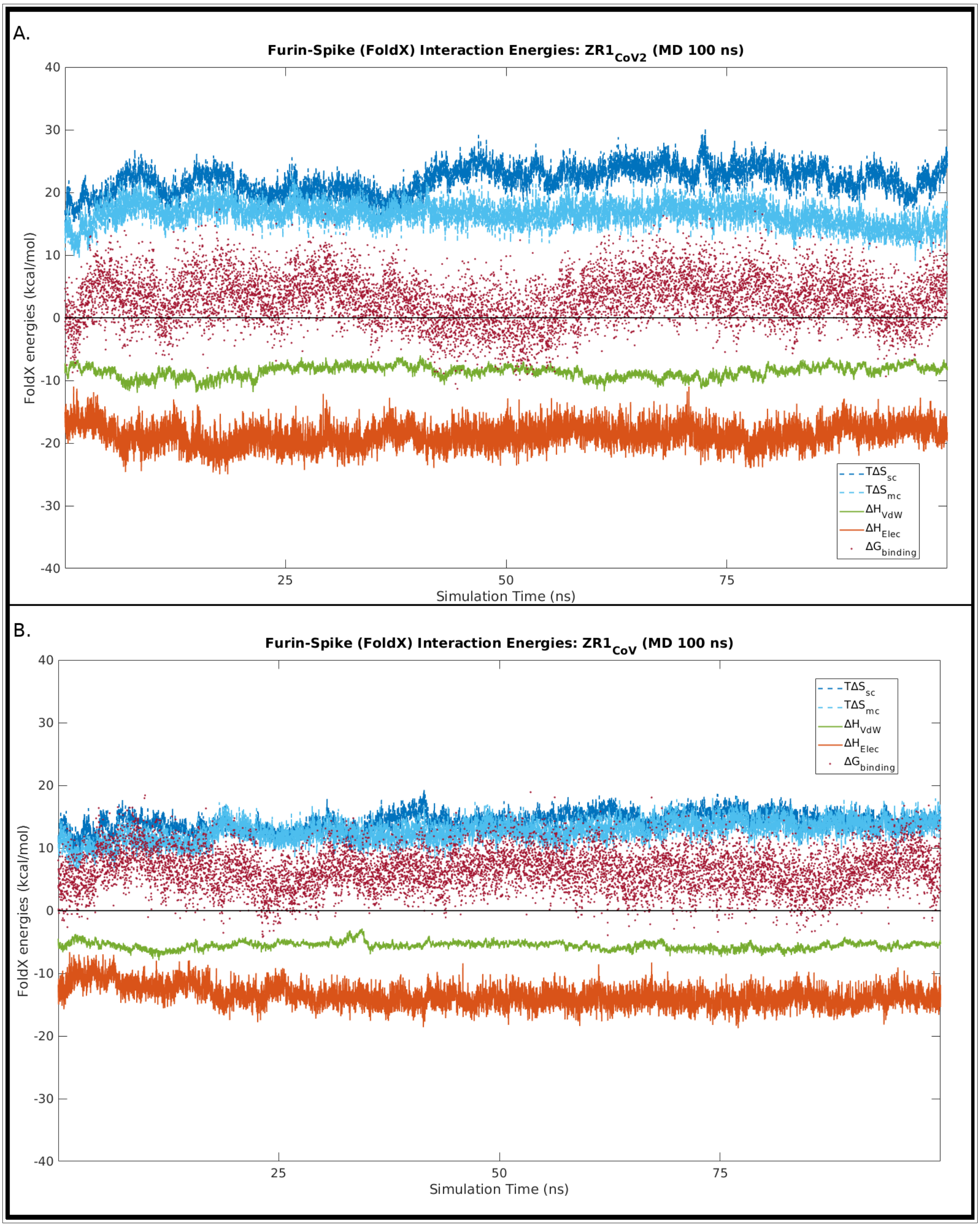

Figure S9. Entropy arrest in the Spike–Furin interactions (SARS-CoV-2, SARS-CoV) as revealed in RR1$_{CoV-2}$, ZR1$_{CoV}$. Entropic energies (in kcal mol^{-1}) are plotted as a function of simulation time (in ns) for RR1$_{CoV-2}$, (300 ns) ZR1$_{CoV}$ (100 ns) respectively. Structural thumbnails in cartoon representation (colored according to chain identities) for Spike, Furin and their complexes are added to each time-series plot as a quick 'at a glance' guide for each subject.

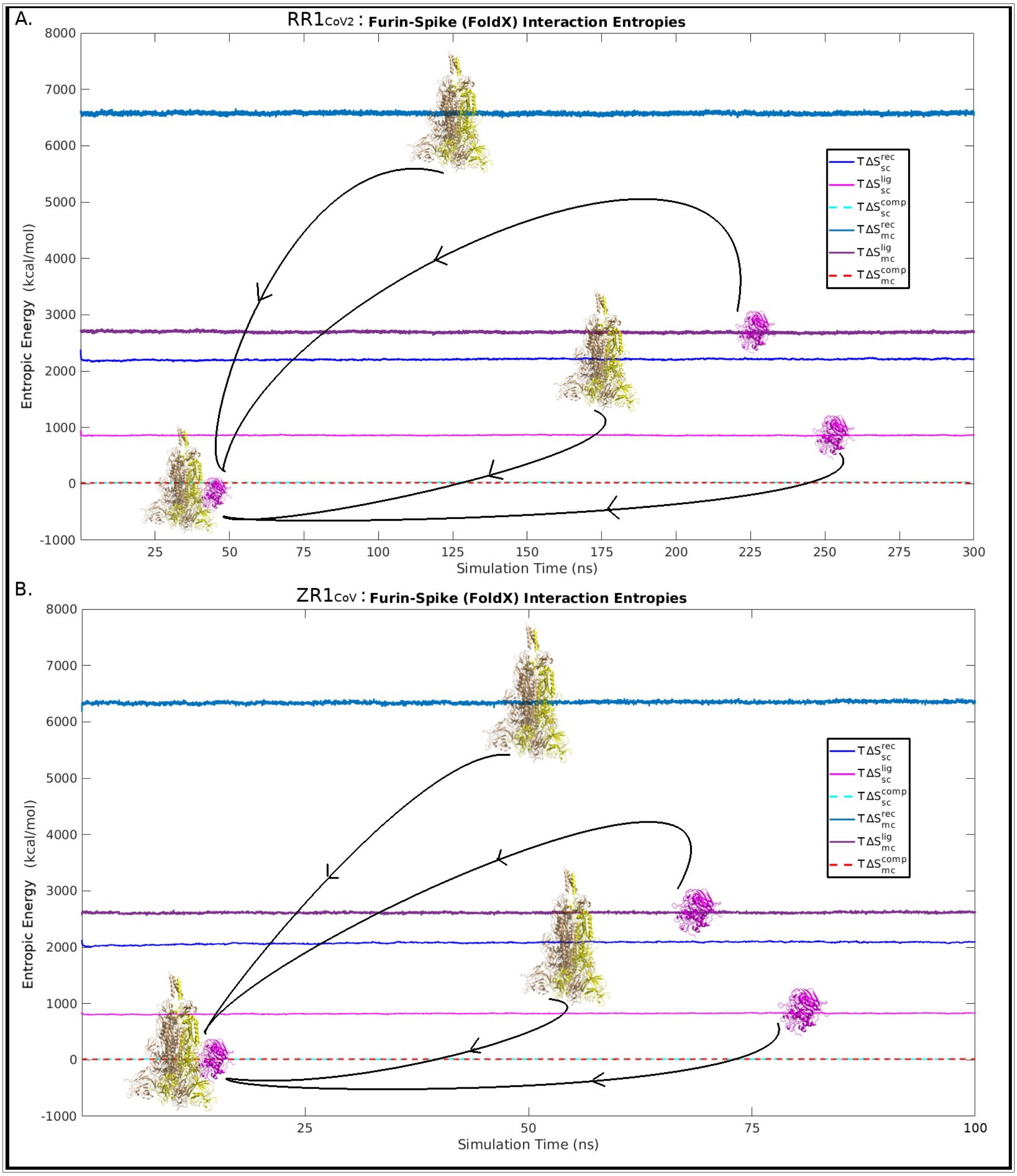

Table S7. Average entropic changes pertaining to the Spike–Furin interaction in $RR1_{CoV-2}$ and $ZR1_{CoV-2}$. The statistics is done over the entire 300 ns trajectory (RR1). Standard deviations given in parentheses. The subscripts in the TΔS terms (in units of kcal mol^{-1}) refer to mc: main-chain, sc: side-chain.

	Receptor (Spike)			Ligand (Furin)			Complex (Spike–Furin)		
	$RR1_{CoV-2}$	$ZR1_{CoV-2}$	$ZR1_{CoV}$	$RR1_{CoV-2}$	$ZR1_{CoV-2}$	$ZR1_{CoV}$	$RR1_{CoV-2}$	$ZR1_{CoV-2}$	$ZR1_{CoV}$
$\langle T\Delta S_{mc} \rangle$	6573.9 (21.8)	6564.5 (23.8)	6344.2 (23.5)	2691.4 (16.1)	2701.4 (14.8)	2613.6 (14.3)	17.9 (2.3)	16.5 (1.9)	12.7 (1.8)
$\langle T\Delta S_{sc} \rangle$	2207.2 (12.9)	2185.1 (14.7)	2077.4 (21.4)	860.8 (6.5)	846.8 (7.4)	821.9 (9.1)	23.4 (3.4)	22.0 (2.2)	14.2 (1.5)

Figure S10. Overlaid Ramachrandran Plots for FLCS$_{Spike}$ pertaining to (A) unbound and (B) Furin-bound Spike states. Each plot is overlaid with 100 atomic models belonging to the same state (unbound or bound). Within each individual plot (i.e., pertaining to each atomic model), the contiguity of {Φ, Ψ} points for successively connected residues in the FLCS$_{Spike}$ loop is portrayed by adding the successively connected residues by thin dashed black lines (--) belonging to the -P$_{681}$-R$_{682}$-R$_{683}$-A$_{684}$-R$_{685}$- pentapeptide motif.

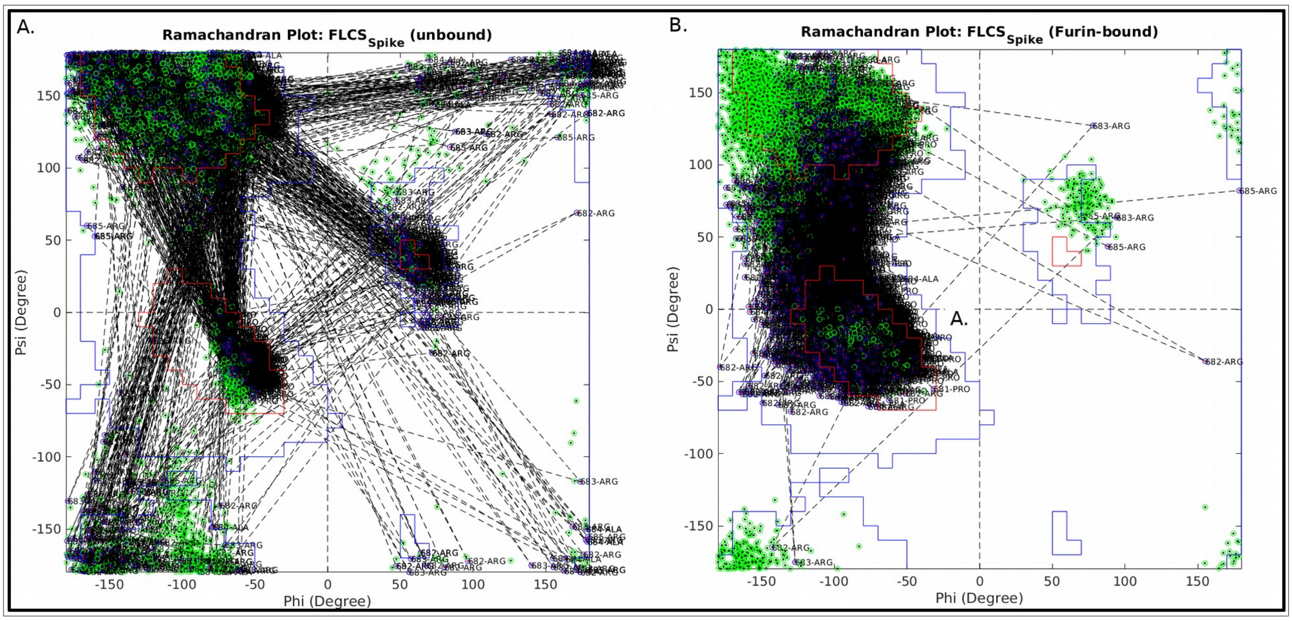

Table S8. The RP-derived parameters for the $FLCS_{Spike}$ patches pertaining to unbound and bound Spike states in SARS-CoV-2. The statistical parameters in the header-cells (mean: $|\delta_c|$, standard deviation: $\sigma(\delta_c)$, median: $<\delta>_{median}$, 3rd quartile: $<\delta>_{3q}$, 9th decile: $<\delta>_{9d}$) are as defined in section **2.7**, **Materials and Methods**.

Spike States	$\lvert\delta_c\rvert$ ($\sigma(\delta_c)$)	$<\delta>_{median}$	$<\delta>_{3q}$	$<\delta>_{9d}$
State-1 (Unbound)	138.5 (30.6)	101.0	212.0	305.2
State-2 (Bound)	118.7 (26.2)	94.7	160.5	196.0
Relative increase from state-1 to state-2 (%)	-16.7	-6.7	-32.1	-55.7

References

1. Edgar, R.C. MUSCLE: A Multiple Sequence Alignment Method with Reduced Time and Space Complexity. *BMC Bioinformatics* **2004**, *5*, 113, doi:10.1186/1471-2105-5-113.
